# Women’s preferences for selective estrogen reuptake modulators: an investigation using the time trade-off technique

**DOI:** 10.1186/2193-1801-3-264

**Published:** 2014-05-24

**Authors:** Angelique F Ralph, Brittany Ager, Melanie L Bell, Ian M Collins, Lesley Andrews, Kathy Tucker, Nicole O’Reilly, Kelly-Anne Phillips, Phyllis Butow

**Affiliations:** School of Psychology, University of Sydney, Sydney, New South Wales 2006 Australia; Psycho-Oncology Co-operative Research Group (PoCoG), University of Sydney, Sydney, New SouthWales 2006 Australia; Mel and Enid Zuckerman College of Public Health, University of Arizona, Tucson, Arizona 85724 USA; Division of Cancer Medicine, Peter MacCallum Cancer Centre, East Melbourne, Victoria 3002 Australia; Sir Peter MacCallum Dept. of Oncology, The University of Melbourne, Parkville, Victoria 3010 Australia; Hereditary Cancer Clinic, Prince of Wales Hospital, 147 Barker Street, Randwick, New South Wales 2031 Australia; School of Psychiatry, University of New South Wales, Randwick, New South Wales 2031 Australia; Centre for Medical Psychology and Evidence-based Decision-making (CeMPED), University of Sydney, Sydney, New South Wales 2006 Australia

**Keywords:** Breast cancer, Chemoprevention, SERMs, Patient preferences, *BRCA1*

## Abstract

**Purpose:**

Selective Estrogen Receptor Modulators (SERMs) reduce the risk of breast cancer for women at increased risk by 38%. However, uptake is extremely low and the reasons for this are not completely understood. The aims of this study were to utilize time trade-off methods to determine the degree of risk reduction required to make taking SERMs worthwhile to women, and the factors associated with requiring greater risk reduction to take SERMs.

**Methods:**

Women at increased risk of breast cancer (*N* = 107) were recruited from two familial cancer clinics in Australia. Participants completed a questionnaire either online or in pen and paper format. Hierarchical multiple linear regression analysis was used to analyze the data.

**Results:**

Overall, there was considerable heterogeneity in the degree of risk reduction required to make taking SERMs worthwhile. Women with higher perceived breast cancer risk and those with stronger intentions to undergo (or who had undergone) an oophorectomy required a smaller degree of risk reduction to consider taking SERMs worthwhile.

**Conclusion:**

Women at increased familial risk appear motivated to consider SERMs for prevention. A tailored approach to communicating about medical prevention is essential. Health professionals could usefully highlight the absolute (rather than relative) probability of side effects and take into account an individual’s perceived (rather than objective) risk of breast cancer.

## Introduction

A strong family history of breast cancer and/or carrying a germline mutation in the *BRCA1* or *BRCA2* gene, substantially increases breast cancer risk (Antoniou et al. 2003; Pharoah et al. 1997). For example, mutation carriers have average lifetime risks of 65% and 45% respectively (Antoniou et al. 2003), compared with 12% for the Australian general population (Australian Institute of Health and Welfare 2009).

Risk reduction strategies for women with an elevated risk of breast cancer include surgery, namely bilateral mastectomy and bilateral pre-menopausal salpingo-oophorectomy, and medication such as selective estrogen receptor modulators (SERMs) (Rebbeck et al. 2009; Rebbeck et al. 2004). There is strong evidence that SERMs such as tamoxifen and raloxifene, taken daily for 5 years, reduce breast cancer risk by 38% (Cuzick et al. 2013). However, uptake of these agents is very low, even in women at high familial-risk (Phillips et al. 2006; Savage 2007; Vogel 2010; Keogh et al. 2009; Evans et al. 2001; Collins et al. 2013). Whilst it has been estimated that 15% of women in the United States aged 35 to 79 could potentially benefit from tamoxifen (Freedman et al. 2003), less than 0.2% of women in this age range are taking tamoxifen (Waters et al. 2010).

The reasons for low SERM uptake are not completely understood, although fear of side effects, difficulty comprehending risk and biases against taking medication, have been shown to be important in several studies (Day et al. 1999; Port et al. 2001; Lovegrove et al. 2000). Understanding how women make decisions about breast cancer risk management might ultimately enhance uptake. The Time Trade-Off (TTO) method, establishes willingness to trade-off quality of life for length of life and has been widely utilized to elicit patients’ preferences in situations that involve complex trade-offs between the benefits and harms of medical decisions (De Haes & Stiggelbout 1996; Duric et al. 2007; Lin et al. 2012; Simes & Coates 2001). Studies investigating patient-preferences for adjuvant-chemotherapy in early breast cancer have found that a surprising number of women judged negligible benefits (0.1% to 1% increase in survival rate) sufficient to make adjuvant-chemotherapy worthwhile (Duric et al. 2007; Simes & Coates 2001). Parenting concerns, minimizing future regret, doubts about information provided by healthcare professionals and feeling they had no choice were the main explanations given by women for judging negligible benefits worthwhile.

The present study aimed to expand on previous research by exploring not only the minimum absolute risk-reduction that women judge necessary to make initiating SERMs worthwhile, but also the clinical and demographic variables associated with this outcome. Based on the literature, we hypothesized that higher objective breast cancer risk, younger age, being a parent and considering risk-reducing surgery would all be associated with requirement of a lower risk reduction to make taking SERMs worthwhile (Lovegrove et al. 2000; Tchou et al. 2004; Jansen et al. 2004).

## Methods

### Participants

Eligibility criteria included: being at moderate or high risk of breast cancer (see below), competency in English, aged 18 to 70 years, and no personal history of breast or ovarian cancer or bilateral mastectomy. Participants who had previously undergone bilateral oophorectomy (which reduces breast cancer risk if done while pre-menopausal) were not excluded, as their residual risk is still high enough to warrant consideration of SERMs.

### Procedure

Consecutive eligible women identified from the clinic databases of two Australian Familial Cancer Clinics (FCCs) were invited to participate by the FCC; interested women were phoned by a researcher to gain verbal consent. Consenting women completed the study online or by paper and pencil. They first completed the consent form, then read a fact sheet that provided information on SERMs, risk-reducing mastectomy and salpingo-oophorectomy to ensure a basic level of knowledge, and then completed the questionnaire. Women were categorized as at moderate or high risk of breast cancer based on family history and *BRCA1* and *BRCA2* mutation status, using Cancer Australia definitions, that is, high risk is greater than three times the population risk and moderate risk is one and a half to three times population risk (NBOCC 2009). Ethics approval for the study was obtained from the University of Sydney and participating sites.

### Measures

#### Demographic characteristics

We collected age, ethnicity, relationship status, parity, number of daughters, plans for future children, menopausal status, *BRCA1 BRCA2* mutation status, cigarette smoking status, personal history of blood clots, and family and/or personal history of bilateral mastectomy and/or oophorectomy and/or SERM use.

#### Intention to take SERMs: patient preferences

The primary outcome, patient preferences, was assessed using the TTO method adapted from (Simes & Coates 2001). Women were presented with hypothetical scenarios and asked to decide between taking and not taking SERMs. Women indicated if they would or would not take SERMs if taking SERMs were to reduce their lifetime breast cancer risk from a starting risk of 50% down to 50% (that is, not reduce their risk at all), then down to 49.5%, to 49%, to 48%, to 45% and so-on down to 0% by 5% increments. A second scenario was presented from a starting risk of 20%, with SERMs hypothetically reducing risk down to 20% (not reducing risk at all) then down to 19.5%, to 19%, to 18%, to 17%, to 15% and so-on down to 0% by increments alternating between 2 and 3%.

These two scenarios were based on the average lifetime risk of developing breast cancer for a woman at high risk (50%) and for a woman at moderate risk (20%). The “tipping-point”, the risk reduction required to make taking SERMs worthwhile, was computed for each participant for both the 50% and 20% risk scenarios by subtracting the percentage where the woman crossed from not taking SERMs to taking SERMs from the corresponding baseline risk. Thus, a lower tipping point represents a lower degree of risk reduction required to intend to take SERMs.

It was emphasized to participants that the choices were hypothetical; there were no right or wrong answers; and the numbers were hypothetical scenarios and did not apply to them personally. Following the TTO, women were asked to list, in order of importance, the three most important factors influencing their decision to take or not take SERMs.

### Data analysis

Associations between TTO tipping points scores and clinical and demographic characteristics were explored through correlations and adjusted-analysis using multiple linear regression. In order to represent the equally important views of women who did not ‘tip’ (i.e., women who consistently chose to either take or not take SERMs, regardless of degree of benefit), 0.5 was added to all tipping point scores. Age and risk status were included as covariates in all multiple linear regressions due to strong theoretical rationale (Lovegrove et al. 2000; Tchou et al. 2004). Intention to have bilateral mastectomy and intention to have (or having had) bilateral oophorectomy were also included as binary covariates, as women do not consider SERMs in isolation but rather as an addition or alternative to other risk reduction strategies (Metcalfe et al. 2007). As history of blood clots and smoking status were not correlated with any of the outcomes, they were not included as covariates. Having a daughter/s was also omitted as a covariate as it demonstrated a large and significant correlation of *r* = .70 (*p* < .001) with another independent variable, parity.

Items women had listed as important in their decision-making during the TTO were thematically analyzed. For each woman, items were weighted from 3 to 1 from highest to least important, and categorized into identified themes. A weighted-frequency score was then calculated for each theme by summing the weightings of each item in the theme.

As some women in the sample had previously taken SERMs, a sensitivity-analysis was conducted to assess the impact this variable had on each of the models.

## Results

### Sample

Of the 407 invitations sent, 117 women responded and 107 (26%) completed the questionnaire. Analysis comparing de-identified data on non-responders with that of responders indicated that more women at moderate risk participated (53%) than did not participate (47%) compared to women at high risk (33% versus 67%) or those who were BRCA1 or BRCA2 positive (24% versus 76%). However as few eligible moderate risk women were identified, the absolute numbers were small. There were no other differences identified between responders and non-responders.

Participant characteristics are summarized in Table 1. The mean age was 43 (*SD* = 10.8). Forty-one women (38% of the sample) were *BRCA1* or *BRCA2* mutation positive, 56 (52%) were at high risk but without a documented mutation and 10 (9%) were at moderate risk.

**Table 1:** **Demographic and clinical characteristics of participants at moderate risk of breast cancer, high risk of breast cancer and those with a mutation in the** ***BRCA1*** **or** ***BRCA2*** **gene**

Variable	Moderate risk		High risk		Mutation positive		Combined	
	***n*** =10		***n=56***		***n*** =41		***N*** =107	
	***M***	***SD***	***M***	***SD***	***M***	***SD***	***M***	***SD***
Age (years)	41.8	10.2	44.5	11.0	41.2	10.7	43.0	10.8
	**n**	**%**	**n**	**%**	**n**	**%**	**n**	**%**
Ethnicity								
Australian	4	40	45	80	30	73	79	74
European	3	30	6	11	4	10	13	12
Asian	0	0	2	4	2	5	4	4
Other	3	30	3	5	5	12	11	10
Relationship status								
Single	1	10	16	29	14	34	32	30
Married/de facto	9	90	40	71	27	66	76	71
Children								
Yes	5	50	11	20	16	39	32	30
No	5	50	45	80	25	61	75	70
Daughter/s								
Yes	4	40	36	64	21	51	61	57
No	6	60	19	34	20	49	45	42
Want future children								
Yes	4	40	10	18	8	20	22	21
No	4	40	40	71	28	68	72	67
Unsure	1	10	6	11	5	12	12	11
Menopausal status								
Premenopausal	7	70	31	55	19	46	57	53
Perimenopausal	1	10	6	11	0	0	7	7
Menopausal	1	10	15	27	20	49	36	34
Unsure	1	10	4	7	2	5	7	7
Family history of prophylactic mastectomy								
Yes	3	30	14	25	17	41	34	32
No	7	70	42	75	24	59	73	68
Family history of prophylactic oophorectomy								
Yes	1	10	10	18	19	46	30	28
No	9	90	46	82	22	54	77	72
Family history of SERMs								
Yes	5	56	26	46	10	24	41	38
No	4	44	30	54	30	73	64	60
Personal history of oophorectomy								
Yes	0	0	8	14	19	46	27	25
No	10	100	48	86	22	54	80	75
Personal history of SERMs								
Yes	1	10	12	21	5	12	18	17
No	9	90	44	79	36	88	89	83
Cigarettes per day								
None	8	80	53	95	40	98	101	94
10 or less	2	20	3	5	0	0	5	5
11 to 20	0	0	0	0	1	2	1	1
History of blood clots								
Yes	1	10	0	0	0	0	1	1
No	9	90	56	100	41	100	106	99

### Tipping points

**Figure 1:**
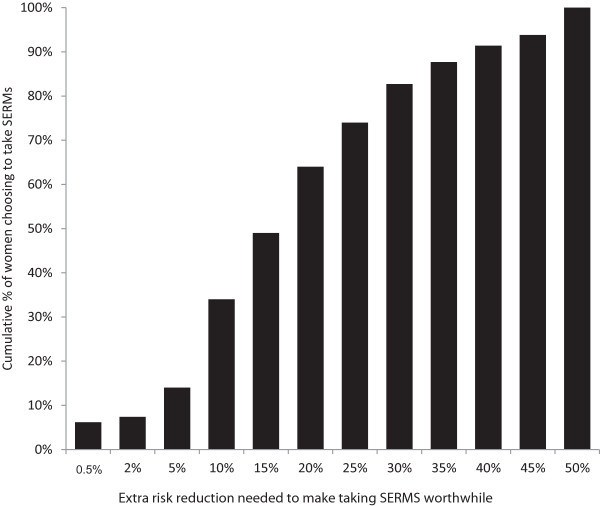
**Amongst women who did ‘tip’ (n = 81), cumulative proportions of women considering taking SERMs for various degrees of risk reduction for 50% baseline scenario.** Twelve participants were excluded from the analysis of the 50%-baseline scenario as they switched multiple times between *taking* SERMs and *not taking* SERMs throughout the same baseline scenario; thus a tipping point could not be calculated.

**Figure 2:**
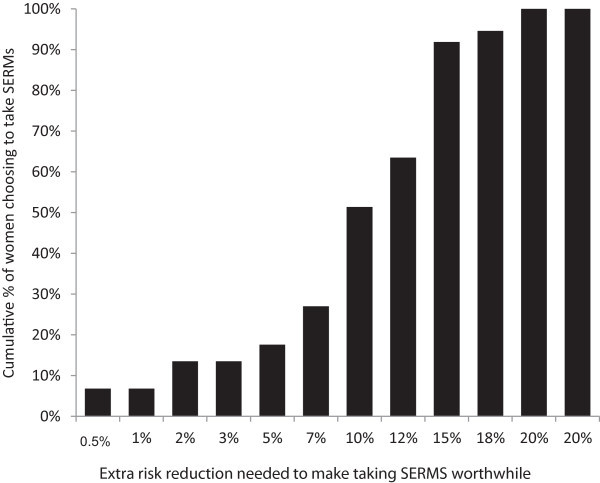
**Amongst women who did ‘tip’ (n = 74), cumulative proportions of women considering taking SERMs for various degrees of risk reduction for 20% baseline scenario.** Eight participants were excluded from the analysis of the 20%-baseline scenario as they switched multiple times between *taking* SERMs and *not taking* SERMs throughout the same baseline scenario; thus a tipping point could not be calculated.

Tipping points are summarized in Figures 1 and 2. Some women had no tipping point. Specifically, 5.4% and 9.2% of women for the 50% and 20% baseline scenarios respectively, invariably chose to take SERMs and 7.5% and 15.3% of women for the 50% and 20% baseline scenarios respectively consistently chose not to take SERMs. There were no significant differences in the mean tipping points between risk groups (i.e., moderate, high, mutation positive) in either the 50%-baseline risk scenario, *F*(2,90) = 2.48, *p* = .09, or the 20%-baseline risk scenario, *F*(2,95) = 1.63, *p* = .20.

Sensitivity analysis showed that having previously taken SERMs did not appreciably change multivariate analysis results, therefore the results shown here include the whole sample. As shown in Table 2, perceived risk and intention to undergo, or has undergone oophorectomy accounted for a significant amount of variance in tipping point scores in both scenarios (*R*^*2*^ = .19, *p* = .01, *R*^*2*^ = .17, *p* = .02 respectively). Women who intended to undergo or had undergone oophorectomy judged smaller amounts of risk reduction sufficient to take SERMs in both the 50% and 20% base risk scenarios. Additionally, women with higher perceived breast cancer risk required a smaller degree of risk reduction for it to be worthwhile for them to take SERMs in the 20% scenario.

**Table 2:** **Multiple linear regression analysis predicting tipping point at 50% and 20% baseline risk**

Dependent variable	Independent variables	B (95% CI)	P-value
Tipping point at 50% baseline risk	(R^2^ = .19)		.01
	Age	.05 (-.3,.4)	.77
	Risk status		
	Moderate	Reference	
	High	-6.9 (-18.3, 4.5)	.23
	Mutation positive	-1.5 (-13.8, 4.5)	.81
	Has children	-2.7 (-10.1, 4.8)	.48
	Intention to have mastectomy	-4.2 (-11.0, 2.6)	.22
	Intention to have oophorectomy	-11.7 (-18.5 ,-4.8)	.001
	Perceived risk	-0.1 (-.15, .13)	.87
Tipping point at 20% baseline risk	(R^2^ = .17)		.02
	Age	.03 (-.1,.2)	.72
	Risk status		
	Moderate	Reference	
	High	-1.5 (-.6.4, 3.4)	.54
	Mutation positive	-.1 (-5.4, 5.2)	.96
	Has children	-.5 (-3.7, 2.7)	.76
	Intention to have mastectomy	-.9 (-3.9, 2.0)	.54
	Intention to have oophorectomy	-3.7 (-6.7, -.8)	.02
	Perceived risk	-.1 (-.1, .0)	.01

Regression coefficients B and 95% confidence intervals (CI) are shown.

### Qualitative responses

Issues affecting their decision, listed by women, are summarized in Table 3. The risk reduction offered by SERMs, and one’s personal level of risk and family concerns, were the most cited reasons for hypothetically choosing to take SERMs. Side effects was clearly the most cited reason for *not* taking SERMs, followed by low perceived efficacy of SERMs and choosing other methods of risk reduction. Most women (*n* = 53) did not specify which of the possible side effects were influencing their decision, however, of those who did, menopausal symptoms was the most frequently reported (*n* = 10), followed by unknown long-term consequences (*n* = 6), sexual function (*n* = 4), fertility (*n* = 4), weight gain (*n* = 2), osteoporosis (*n* = 3) and cognition (*n* = 1).

**Table 3:** **Categories of factors women considered most influential in their hypothetical decision to take or not take SERMs**

Category		Weighted frequency ^a^
Incentives		
	Risk reduction	96
	Personal risk	52
	Reduce stress and worry	17
	Family concerns	25
	Age	2
	Side effects^b^	2
Barriers		
	Side effects	214
	Other risk reduction methods	46
	Inadequate efficacy	140
	Taking medication	29
	Age	15
	Lack of information	10
	Medical advice	18
	Cost	14

^a^Weighted frequency was calculated by giving weightings of 3, 2 and 1 to answers listed as of highest importance, of next degree of importance and of least importance respectively. ^b^Positive side effects were listed.

## Discussion and conclusion

### Discussion

This study is the first to our knowledge to apply the Time Trade-Off (TTO) method to elucidate how women at increased risk of breast cancer make decisions regarding using SERMs to reduce risk. The substantial minority of women who had no tipping-point was surprising. Six percent and 16% of women, for the 50% and 20% baseline scenarios respectively, consistently chose to take SERMs even when there would not be any reduction in the risk of breast cancer. These women reported being most influenced by: minimizing the stress and worry associated with being at increased risk; family concerns; and lowering their risk of breast cancer. Previous research has found that many women judge negligible benefits sufficient to engage in treatment for breast cancer (Duric et al. 2007; Simes & Coates 2001; Heisey et al. 2006). For example, Duric and colleagues found that 52-61% of women with early stage breast cancer judged one extra day in a life expectancy of 5-years sufficient to have adjuvant chemotherapy, regardless of the baseline risk, of breast cancer related death (Duric et al. 2007).

Further, many women would consider SERMs if the benefits were sufficiently large, with 70% of moderate and 90% of high risk women respectively willing to consider SERMs for a 40% or less risk reduction (likely realistic). Thus general interest in SERMs in this group of moderate and high risk women was high.

A small proportion of women consistently chose not to take SERMs, even if SERMs reduced their risk of breast cancer to zero. All such women listed side effects as one of the top three important factors influencing their decision. This is congruent with previous research (Port et al. 2001; Lovegrove et al. 2000; Bober et al. 2004) demonstrating that women often decline to take tamoxifen because of fear of side effects which are frequently overestimated. Future research should explore which side effects are most concerning to women. Perhaps women could be offered a trial of SERMs to determine if they are substantially affected by vasomotor and gynecologic side effects, before making a decision whether to plan for 5 years of use. Communicating absolute, rather than relative risks for serious potential side effects such as endometrial cancer and thrombosis may also help to put these into perspective, especially for pre-menopausal women where they are rare (Keogh et al. 2009; Harvey et al. 2011; Fisher et al. 1998).

Unlike low benefits required by most women with breast cancer to take adjuvant chemotherapy (Duric et al. 2007), many women in the current study required a large risk reduction before choosing to take SERMs. In the 20% base risk scenario, over 40% of women needed the risk to be halved to 10% in order to consider taking SERMs to be worthwhile. Previous studies have reported that healthy individuals with no current symptoms have a lower tolerance for potential toxicities. Therefore, women ‘at risk’ rather than ill may require greater potential benefit from SERMs to outweigh the associated negative aspects and increase the likelihood of choosing this risk reduction option (Lawrence et al. 2012).

Younger age, higher objective risk and having children produced results in the predicted direction, however, none were significantly associated with degree of risk reduction required to take SERMs, for either the 20% or the 50% baseline risk scenario. Previous studies have had conflicting findings regarding the association between age and acceptance of risk-reducing tamoxifen (Lovegrove et al. 2000; Tchou et al. 2004; Bober et al. 2004; Meiser et al. 2003). Whilst some women may decline SERMs to avoid premature menopausal symptoms, other women may be more influenced by the fact that the most favorable risk benefit ratio is seen in premenopausal women (Harvey et al. 2011).

Women who reported that they were likely to undergo (or had undergone) an oophorectomy required less risk reduction in order to choose to take SERMs than women who were not considering this procedure. It is perhaps not surprising that women who are motivated enough to consider surgical risk reduction may be more willing to accept SERMs.

Subjective but not objective breast cancer risk was predictive of the degree of risk reduction required to take SERMs in the 20% baseline condition, with neither predictive in the 50% baseline condition. This is congruent with previous studies where subjective but not objective risk was associated with either considering tamoxifen (Meiser et al. 2003) or uptake of tamoxifen (Tchou et al. 2004; Bober et al. 2004). It is a well documented phenomenon that women overestimate their risk of breast cancer e.g. (Lovegrove et al. 2000; Heisey et al. 2006; Black et al. 1995; Davis et al. 2004; Lerman et al. 1995) and that perception of risk is rarely a direct comprehension of accurately understood probability information (Bober et al. 2004; Hopwood 2000). Thus care must be taken in discussing risk with these women, as it will influence their decision-making.

The present study has a number of limitations. It was cross-sectional and hypothetical, thus causation cannot be implied and it is not known whether intention to take SERMs would translate into actual behavior. Furthermore, women are unlikely to consider SERMs in isolation but rather as an addition or alternative to other methods of risk reduction such as bilateral mastectomy and/or oophorectomy. However, it was not feasible in the current study to examine women’s preferences for multiple methods of risk reduction.

The study had a relatively low response rate of 26%, although data on non-responders suggests this did not result in a biased sample. Nevertheless, the relatively high prevalence of previous SERM use in this sample, compared with expected rates, (Collins et al. 2013) suggests that the sample could have been unusual, thus results should be generally applied with some caution. Women who attend Australian familial cancer clinics have above-average educational and socioeconomic levels and may not be representative of the broader population of women at increased risk (Meiser et al. 2000; Coyne & Anderson 1999; Coyne et al. 2000; Cull et al. 1998). Nonetheless, findings are highly relevant to countries such as Australia, where the vast majority of assessment and genetic-testing of women at increased familial risk is done by a network of Family Cancer Centers, and these women are the most likely to be offered SERMs (Keogh et al. 2009).

### Practice implications and future research

Many women at increased risk of breast cancer are interested in using SERMs to reduce their risk. Health professionals could focus on informing women about the proven long-term benefits of SERMs and the high quality of the underpinning evidence. As side effects represent a highly salient factor to women, and are often overestimated, the absolute (rather than relative) probability of side effects should be highlighted. Our study shows that individual preferences vary widely and thus a tailored approach to medical prevention is essential, perhaps with the assistance of a computerized decision aid that can effectively translate reported relative benefits and risks into absolute benefits and risks individualized to each woman’s circumstances.

Future research should endeavor to assess women’s preferences for SERMs in the context of other risk-reduction methods and/or in a sample of women who have declined surgical risk reduction procedures.

### Ethical standards

This study was carried out according to national legislation and was approved by the Human Research Ethics Committee (12/020 [HREC/12/POWH/42]).
